# The association between depressive symptoms and fall accidents among middle-aged and elderly people in China

**DOI:** 10.1186/s12199-018-0735-y

**Published:** 2018-09-05

**Authors:** Peng Ouyang, Wenjun Sun

**Affiliations:** School of Management, Harbin Institute of Technology, 92 West Dazhi Street, Nan Gang District, Harbin, Heilongjiang Province People’s Republic of China

**Keywords:** Fall accidents, Depressive symptoms, CHARLS, CES-D

## Abstract

**Background:**

Depressive symptoms are a worldwide health problem. However, the research about the effect of depressive symptoms on the fall among the Chinese mid-aged and elderly people is lacking. Therefore, this study aims to investigate the association between depressive symptoms and fall accidents among middle-aged and elderly people in China.

**Methods:**

This study was conducted based on 12,527 sets of data from China Health and Retirement Longitudinal Survey (CHARLS). The 2011 depressive symptoms data and the 2013 fall data were chosen for this study. The depressive symptom-related data was assessed by the Chinese version of Center for Epidemiologic Studies Depression scales (CES-D). Individuals were divided into subgroups according to gender (male or female), age (45–59, middle-aged or ≥ 60, elderly people), and residence (rural or urban). The odds ratios (ORs) were compared between subgroups using multivariable logistic regression analysis method.

**Results:**

The adjusted OR value (OR = 1.19 [95% CI 1.07–1.33]) shows there is a significant association between depressive symptoms and subsequent fall accidents. The ORs of the female, elderly people, rural, and urban subgroups are 1.31 (95% CI 1.11–1.55), 1.24 (95% CI 1.08–1.43), 1.17 (95% CI 1.02–1.33), and 1.25 (95% CI 1.04–1.49), respectively, which reveals that this association is also statistically significant in these subgroups.

**Conclusions:**

This study shows that there is a significant association between depressive symptoms and their subsequent fall accidents among the Chinese middle-aged and elderly people.

## Background

Depressive symptoms are a kind of common disorder which is characterized by sadness, loss of interest in activities, and decreased energy and may occur throughout the life-course [1, 2]. They have become a serious health issue around the world in both developed and developing countries. It is estimated that 322 million people were suffering from depressive symptoms in the worldwide [3]. Depressive symptoms are the fourth cause of disability around the world and are projected to be the second leading cause by 2020 [4]. Depressive symptoms are often associated with health issues such as chronic pain [5] and impaired quality of life [6].

Fall and its related injuries are expected to be the leading cause of morbidity and disability worldwide. Fall can cause adverse physical consequences such as fractures, restriction of active ability, deterioration of health, and decreased physical activity as well as reduced psychosocial consequences such as social isolation, low mode, and risk of institutionalization [7, 8]. Moreover, fall would have an important impact on subsequent disability, quality of life, and mortality [9, 10].

Both depressive symptoms and fall are important health issues. It should be noted that depressive symptoms and fall are both dynamic and under progressive processes. Previous studies indicated that fall can result in depressive symptoms, and in turn, depressive symptoms can lead to fall accidents, which can further cause many health problems [11–14]. Previous literature have studied the association between baseline depressive symptoms and subsequent fall accidents [11–15] in other populations; however, the research relating to the association between depressive symptoms and subsequent fall accidents for Chinese people aged 45 and over is lacking. Since 30% of men and 43% of women in China were suffering from depressive symptoms [16], it is urgent and meaningful to investigate the association between depressive symptoms and fall accidents. Moreover, a better understanding of the association between depressive symptoms and their subsequent fall accidents is highly valuable for fall prevention from public health perspective. It is supposed that depressive symptoms can lead to higher risk of fall accidents in the following life. Hence, the possible contribution of depressive symptoms on fall accidents among the Chinese mid-aged and elderly people who have depressive symptoms is investigated in this study.

## Methods

### Study design and participants

This was a cohort study. The data was obtained from the China Health and Retirement Longitudinal Study (CHARLS) [16, 17]. CHARLS is designed to collect a high-quality data about people aged 45 and older in China, which aims to be nationally representative and open to the public. CHARLS is a longitudinal survey beginning in 2011, which provides seven parts of information about the respondents: (1) demographic background; (2) family information; (3) health status and functioning; (4) health care and insurance; (5) work, retirement, and pension information; (6) income, expenditure, and assets; and (7) interviewer observation. Individuals will be followed up every 2 years. The institutional review board at Peking University has approved the data collection, and the data will be updated annually. More details about CHARLS can be found on the official website: http://charls.pku.edu.cn/en. In this research, the respondents were chosen from people who were 45 and older. CHARLS 2011 baseline data were used to calculate the Center for Epidemiologic Studies Depression (CES-D) score while other variates were obtained from CHARLS 2013 survey data including fall assessment. There were 18,175 people aged 45 and over in 2011 data, 13,648 of them provided the depression data in 2011, and 13,632 of them provided response to fall data in 2013. The drop rate (< 0.12%) was low for both key variables. It is a pity that in 2011 4527 of them did not provide the depression data. Therefore, we did not include this part of population. We discarded 1115 of them due to the missing data of other variables. Finally, we included 12,527 people in our paper.

### Measures

Depressive symptom was assessed based on the Chinese version of scale items developed by the Center for Epidemiologic Study. There were ten questions with a scale of four points (CES-D 10). Cronbach’s *α* of the Chinese version of CES-D reached 0.815, which indicated the validity and reliability [16]. The CES-D score of 10 was the cut-off point of depressive symptom diagnosis.

Fall accident was evaluated based on the following single item: “have you ever experienced fall accidents in the last two years”. This item was rated on a 0–1 scale. We tested the validity of self-reported fall results based on a similar method used in the literature [18] which used self-reported balance performance for this test. For balance measurement, we used two methods: semi-tandem and full-tandem. Semi-tandem test is whether the person can stand with the side of the heel of one foot touching the big toe of the other foot for about 10 s. If the participants could complete the test without assist, we consider it is a good status of balance; otherwise, it is poor. Full-tandem test is whether the participant could stand with the heel of one foot in front of and touching the toes of the other foot for about 30/60 s. The 30 s was for people who were 70 years old or above, and the 60 s was for people who were less than 70 years old. The evaluation is similar to semi-tandem test evaluation. We calculated the difference of fall for people with different performance levels of balance. As showed in Table 1, the difference of fall for people with different levels of balance was highly significant (*p* <  0.05). Fall was significantly associated with balance assessment. Thus, self-reported fall result has enough validity.

**Table 1:** Validity of self-reported fall among mid-aged and elderly people in CHARLS

	Balance performance (full-tandem test, *N* = 9277)		
	Poor (*N* = 2334)	Good (*N* = 8105)	*p* value
Fall	19.5%	14.5%	< 0.001
	Balance performance (semi-tandem test, *N* = 12,207)		
	Poor (*N* = 180)	Good (*N* = 10,550)	*p* value
Fall	25%	15.7%	0.001

Participants were also required to provide the following information including age, gender, marital status, education level, family members, alive children, residence, annual income, activities of daily living (ADL), chronic disease status, smoking, drinking, and sleep time which were used as covariates. Cohabited was considered as married while separated, divorced, or widowed were defined as unmarried. Old-style private school was regarded as the same level with elementary school. The living condition can be obtained from the place of residence information, which was divided into the rural area or the urban area. ADL was used to evaluate the respondent’s physical performance. The respondents were surveyed with the following question: do you have any difficulty with dressing, bathing or showering, eating such as cutting up food, getting into or out bed, controlling urination, and defecation? The score of 0 suggests that the respondent did not report any problems with the above activities while the score of 1 indicates the respondent had difficulty in finishing any of these activities. The ADL score is the summary score of the above items. Turning to the chronic disease status, the quantity of chronic diseases diagnosed by a doctor for each respondent was calculated. There were 14 types of chronic diseases on the answer list in the CHARLS: (1) hypertension; (2) dyslipidemia; (3) diabetes or high blood sugar; (4) cancer or malignant tumor; (5) chronic lung diseases; (6) liver disease; (7) heart disease; (8) stroke, (9) kidney disease; (10) stomach or other digestive disease; (11) emotional, nervous, or psychiatric problems; (12) memory-related disease; (13) arthritis or rheumatism; and (14) asthma. The base value for chronic disease status was 0, and the maximum was 14. For smoking status, we concentrated on whether they smoked ever while for drinking status, we focused on whether they drunk any alcohol last year. People with depressive symptoms suffered from high prevalence of sleep disorder [19] while most fall accidents occurred during sleep. In this research, we also included sleep time as covariate variable. Sleep time was evaluated based on this question: “During the past month, how many hours of actual sleep did you get at night (average hours for one night, this may be shorter than the number of hours you spent in bed).”

### Statistical analyses

The means and standard deviation were used to describe continuous variable data, and percentage was used to display the categorical variables data. Individuals were allocated into two groups based on the CES-D score (≥ 10 and < 10). The difference between the two groups was compared through the *t* test method for continuous variables and the chi-square test for categorical variables. The depressive symptoms data was from the baseline CES-D score, which was obtained from the 2011 CHARLS survey data, and other variable data were extracted from the 2013 CAHRLS survey data.

The analysis was performed using a series of logistic regression model based on the baseline CES-D score and other covariates as predictor variables. The odds ratio (OR) and 95% confidence interval (CI) were calculated. A receiver operating characteristic curve (ROC) was plotted, and the area under the ROC curve (AUC) was calculated to evaluate the model’s performance. Age and gender were adjusted in the first model (model 1), and age, gender, marital status, education level, household members, alive children, place of residence, annual income, ADL, smoking, drinking, and sleep time were adjusted in the second model (model 2).

The difference of the subgroups (age, gender, and place of residence) was investigated. As mentioned above, participants were divided based the gender (male or female), age (mid-age: < 60 or elderly people: ≥ 60), and residence (rural and urban). In each model, the participant whose CES-D score was less than 10 was selected as reference. In the subgroup analysis, gender was excluded in the analysis of gender subgroup and place of residence was also excluded in the analysis for place of residence subgroups.

If the *p* value is less than 0.05, it will be considered as statistically significant. All of the work was conducted using the Stata of version 13.

## Results

### General characteristics of the study population

Table 2 shows the general characteristics of the participants in this study. The average age of the participants is 60.5 ± 9.2 years (61.3 ± 9.2 years in the group with depressive symptoms individuals and 60.0 ± 9.2 years in normal group). The proportion of participants with depressive symptoms is 36.8%, and the proportion of the participants who have experienced fall accidents is 16.3%. After the participants were separated according to the CES-D score, the fall prevalence of the people suffered from depressive symptoms is significantly higher than that of the normal people (*p* < 0.01). The proportion of participants who suffered from depressive symptom is 20.7%, and the percentage for normal participants is 13.7%. Similar to fall accidents, people whose CES-D score is 10 points or more have more difficulties in ADL than participants whose CES-D score is less than 10 (0.6 ± 1.2 in depressive symptoms group and 0.2 ± 0.6 in normal group, *p* < 0.01). Statistical data shows that male people are more likely to suffer from depressive symptoms. The people living in rural areas are also more likely to suffer from depressive symptoms. In addition, the percentage of people with less education is higher when compared with people who have received good education. There is no noticeable difference between the subgroups divided based on the household members (*p* = 0.473).

**Table 2:** Comparison of general characteristics of cases with CES-D ≥ 10 and CES-D < 10

	Total (*N* = 12,527)	CES-D ≥ 10 (*N* = 4607)	CES-D < 10 (*N* = 7920)	*p* value
Age	60.5 ± 9.2	61.3 ± 9.2	60.0 ± 9.2	< 0.001
Gender (male)	52.8%	62.1%	47.4%	< 0.001
Marital status (married)	87.1%	82.5%	89.8%	< 0.001
Education				
Illiteracy	44.9%	55.2%	38.9%	< 0.001
Elementary school	22.5%	22.4%	22.6%	0.733
Middle school	20.9%	16.0%	23.8%	< 0.001
High school and above	10.1%	5.9%	12.5%	< 0.001
College and above	1.6%	0.5%	2.2%	< 0.001
Place of residence (rural)	62.4%	69.2%	58.2%	< 0.001
Household members	3.6 ± 1.8	3.6 ± 1.8	3.6 ± 1.8	0.473
Alive children	2.7 ± 1.4	2.9 ± 1.5	2.6 ± 1.4	< 0.001
Annual income (RMB)				
≤ 20,000	39.2%	47.2%	34.7%	< 0.001
(20,000, 30,000)	8.3%	8.1%	8.4%	0.555
(30,000, 50,000)	35.7%	34.4%	36.4%	0.028
> 50,000	16.8%	10.3%	20.5%	< 0.001
ADL	0.3 ± 0.9	0.6 ± 1.2	0.2 ± 0.6	< 0.001
Chronic disease status	1.9 ± 1.6	2.4 ± 1.8	1.6 ± 1.5	< 0.001
Drinking (last year, yes)	33.6%	27.7%	37.1%	< 0.001
Smoking (ever, yes)	42.6%	37.8%	45.3%	< 0.001
Sleep time	6.2 ± 1.9	5.7 ± 2.0	6.4 ± 1.7	< 0.001
CES-D score	8.4 ± 6.3	15.2 ± 4.5	4.4 ± 2.8	< 0.001
CES-D ≥ 10	36.8%			
Fall accidents (yes)	16.3%	20.7%	13.7%	< 0.001

Continuous variables are presented as mean ± standard deviation, and categorical variables are displayed as percentage

*ADL* activity of daily living

### Depressive symptoms and fall accidents

**Fig. 1:**
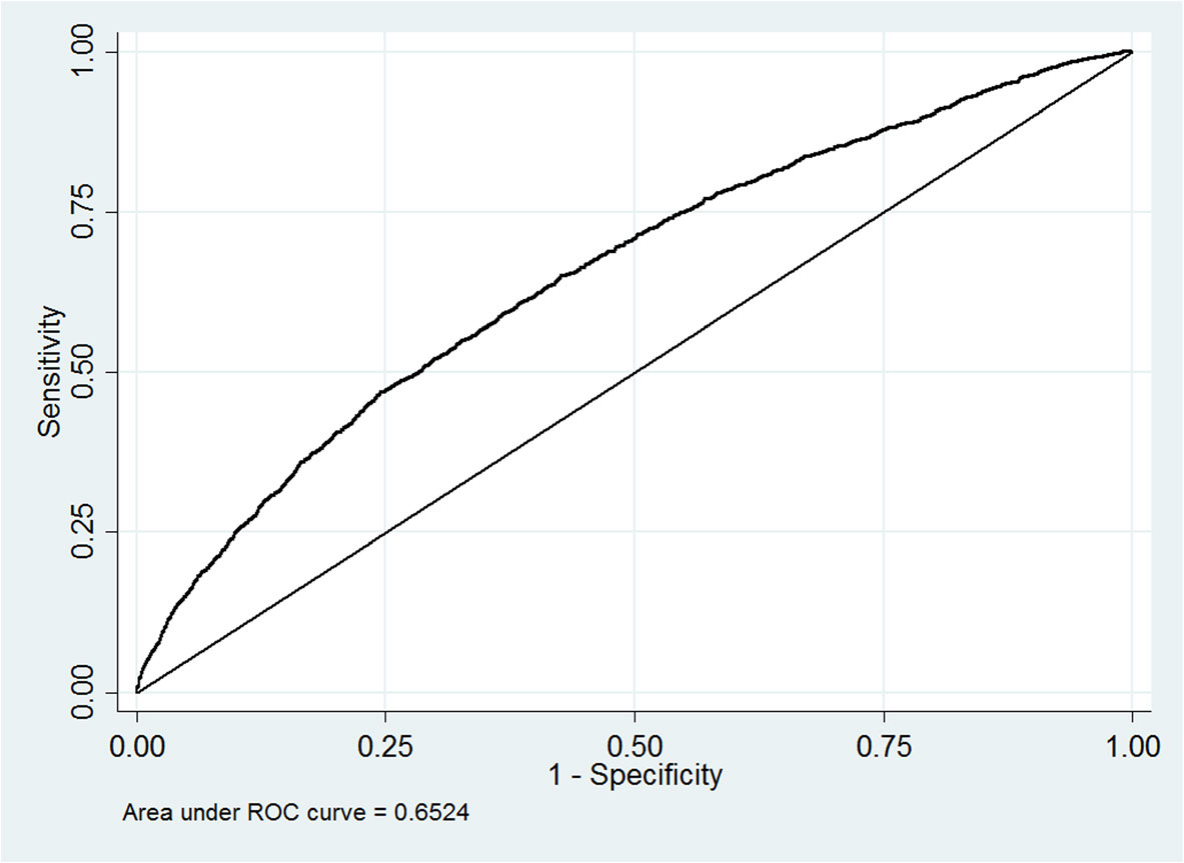
ROC curve of model 2 for all participants

Figure 1 shows the roc curve. The AUC is 0.652, which is larger than 0.5, which shows a good performance of the logistic model. Table 3 (CES-D score as continuous variable) and Table 4 (CES-D score with 10 as cut-off value) present the results of logistic regression analysis of depressive symptoms and fall accidents. Table 3 shows that a higher CES-D score is significantly related to higher likelihood of fall accidents in all models. It can be seen from the OR values in Table 4 that the occurrence of subsequent fall accidents is positively correlated with depressive symptom. After being adjusted with age and gender (model 1), the OR value is lower (adjusted OR 1.46 [95% CI 1.28–1.65]). Although the OR value becomes lower after being adjusted with all of the covariates, the existence of the association between depressive symptoms and its subsequent fall accidents can still be shown (adjusted OR 1.19 [95% CI 1.07–1.33]). When it comes to the subgroups divided according to gender, the OR value of the female subgroup shows a significant association while the male subgroup does not. Moreover, the OR values of the female subgroup is higher than that of the male in all models. In addition, the OR values of the subgroups divided according to the place of residence show statistically significant associations. The OR value of the subgroup of the elderly people is higher than that of the middle-aged people in all models, but the OR value in mid-aged people does not show significant association. Also, the OR value of the subgroup composed with individuals who lived in an urban area is higher than that of people who lived in a rural area in all of the three models.

**Table 3:** Logistic regression analysis of depressive symptoms (CES-D score as continuous variable) and subsequent fall accidents among subgroups

	Unadjusted model OR (95% CI)	Model 1 OR (95% CI)	Model 2 OR (95% CI)
All participants (*N* = 12,527)	1.05 (1.04–1.05)	1.04 (1.03–1.05)	1.02 (1.01–1.03)
	*p* < 0.001	*p* < 0.001	*p* < 0.001
Gender subgroups			
Male (*N* = 6614)	1.04 (1.03–1.05)	1.04 (1.03–1.05)	1.01 (1.00–1.02)
	*p* < 0.001	*p* < 0.001	*p* = 0.027
Female (*N* = 5913)	1.05 (1.04–1.07)	1.05 (1.04–1.06)	1.03 (1.02–1.05)
	*p* < 0.001	*p* < 0.001	*p* < 0.001
Age subgroups			
Mid-age (45–59) (*N* = 6184)	1.04 (1.03–1.05)	1.04 (1.03–1.05)	1.02 (1.00–1.03)
	*p* < 0.001	*p* < 0.001	*p* = 0.009
Elderly people (> = 60) (*N* = 6343)	1.05 (1.04–1.06)	1.04 (1.03–1.05)	1.02 (1.01–1.03)
	*p* < 0.001	*p* < 0.001	*p* < 0.001
Place of residence subgroups			
Rural (*N* = 7800)	1.05 (1.04–1.06)	1.04 (1.03–1.05)	1.02 (1.01–1.03)
	*p* < 0.001	*p* < 0.001	*p* < 0.001
Urban (*N* = 4727)	1.05 (1.04–1.06)	1.04 (1.03–1.06)	1.02 (1.00–1.03)
	*p* < 0.001	*p* < 0.001	*p* < 0.001

*OR* odds ratio, *CI* confidence interval

Unadjusted model: CES-D score alone

Model 1 covariates: age, gender

Model 2 covariates: age, gender, marital status, education level, household members, alive children, place of residence, annual income, chronic disease status, ADL, smoking, drinking, and sleep time

**Table 4:** Logistic regression analysis of depressive symptoms (CES-D ≥ 10) and subsequent fall accidents among subgroups

	Unadjusted model OR (95% CI)	Model 1 OR (95% CI)	Model 2 OR (95% CI)
All participants (*N* = 12,527)	1.65 (1.50–1.82)	1.55 (1.40–1.70)	1.19 (1.07–1.33)
	*p* < 0.001	*p* < 0.001	*p* = 0.001
Gender subgroups			
Male (*N* = 6614)	1.52 (1.34–1.73)	1.46 (1.28–1.65)	1.12 (0.98–1.28)
	*p* < 0.001	*p* < 0.001	*p* = 0.108
Female (*N* = 5913)	1.71 (1.47–1.99)	1.68 (1.44–1.96)	1.31 (1.11–1.55)
	*p* < 0.001	*p* < 0.001	*p* = 0.001
Age subgroups			
Mid-age (45–59) (*N* = 6184)	1.52 (1.31–1.76)	1.48 (1.27–1.71)	1.14 (0.97–1.34)
	*p* < 0.001	*p* < 0.001	*p* = 0.109
Elderly people (> = 60) (*N* = 6343)	1.69 (1.49–1.92)	1.60 (1.40–1.82)	1.24 (1.08–1.43)
	*p* < 0.001	*p* < 0.001	*p* = 0.003
Place of residence subgroups			
Rural (*N* = 7800)	1.63 (1.44–1.83)	1.53 (1.35–1.73)	1.17 (1.02–1.33)
	*p* < 0.001	*p* < 0.001	*p* = 0.024
Urban (*N* = 4727)	1.72 (1.46–2.02)	1.61 (1.37–1.90)	1.25 (1.04–1.49)
	*p* < 0.001	*p* < 0.001	*p* = 0.016

*OR* odds ratio, *CI* confidence interval

Unadjusted model: CES-D score ≥ 10 alone

Model 1 covariates: age, gender

Model 2 covariates: age, gender, marital status, education level, household members, alive children, place of residence, annual income, chronic disease status, ADL, smoking, drinking, and sleep time

## Discussion

To our knowledge, this study is the first to investigate the association between depressive symptoms and the fall accidents among the Chinese mid-aged and elderly population based on a national sample population. There are three major findings in this study: first, there exists a significant association between depressive symptoms which is assessed by CES-D and fall accidents among the middle-aged and elderly people in China; second, the results of the subgroups divided according to the place of residence show that statistically significant association also exists within this subgroup; third, for gender and age subgroups, the statistically significant association exists in female and elderly people, and others does not exist.

Depressive symptom is becoming an important health concern in China. Former studies have noticed the high prevalence of depressive symptoms among Chinese adults. Estimation based on a National Household Survey showed that the prevalence rate of depressive symptoms among the Chinese adult population was 37.9% [20]. One study from CHARLS indicated that the prevalence of depressive symptoms among Chinese mid-aged and elderly people reached 36.7% [16]. Another study from CHARLS suggested the same high-level prevalence of depressive symptoms which reached 36.25% [21]. Our study did not show much difference on prevalence of depressive symptoms with others. The prevalence of depressive symptoms in our study was 36.8%. This was in line with other studies. Some studies from parts of China for example Zoucheng County of Shandong province showed a littler lower level of depressive symptoms, but still reached 27.4% [22]. Considering the negative outcomes caused by depressive symptoms, the high prevalence of depressive symptoms highlighted the importance of our study.

Previous studies have indicated the existence of association between depressive symptoms and fall accidents. Most of the studies demonstrated the existence of association between depressive symptoms and fall accidents except for the study conducted by Kamińska et al. In their study, it is showed that depressive symptoms assessed by geriatric depression scale (GDS) were not related to the occurrence of falls [23]. Study showed that depressive symptoms assessed by GDS increased the risk of fall accidents [24], which was also proven by Brito et al. [25]. Results of the study conducted by Sheeran et al. showed that the fall accidents of the patients with depressed mood were nearly three times higher than that of the normal people [26]. A recent study from the Health and Retirements Study suggested that depressive symptoms was associated with an increased in fall risk 2 years later in America community-dwelling adults ages ≥ 65 [15]. In our study, we found that there is a significant association between depressive symptoms and its subsequent fall accidents among Chinese mid-aged and elderly population, as well as specific subgroups. The results of this study are consistent with most of the previous studies [15, 24, 26]. The difference of this study from other studies is the assessment of depressive symptoms. Most of the former studies assessed depressive symptoms using the GDS. In recent studies, the CES-D has been widely used to evaluate the depressive symptoms because of its good validity and reliability [16, 27, 28]. Besides, it is also found that depressive symptoms are more prevalent among the elderly people compared with mid-aged people. Hence, the difference of the study results may be due to the difference of the population composition.

Many studies have tried to reveal the mechanism of the association between depressive symptoms and its subsequent fall accidents; however, there is no definitive conclusion. Brito et al. proposed that the fall accident is most likely due to the side effect of the drugs in the treatment of the depressive symptoms [25]. In some studies, the greater risk of fall accidents is attributed to the treatment of depressive symptoms, such as the use of benzodiazepine and serotonin reuptake selective inhibitors [25, 29]. A critical systematic review found that psychotropics such as benzodiazepines, antidepressants, and antipsychotics may increase the risk of fall accidents [30]. Kerse et al. found that both the depressive symptoms with clinically significant symptoms and the antidepressants especially for selective serotonin reuptake inhibitors (SSRI) were associated with falls [31]. Therefore, drugs used in the treatment of depressive symptoms may cause the fall accidents in this study. Another explanation of the association between depressive symptoms and their subsequent fall accidents is that postural abnormalities is associated with depressive symptoms when standing, which may increase the risk of fall accidents [32]. In this study, the association cannot be contributed to these two inferences since the medication data were not collected in the CHARLS and the information of the postural abnormalities is lacking. Further research is needed in the future.

Importantly, our results can be used as reference to prevent people suffering from depressive symptoms in China. Except for an effort on declining depressive symptoms, the current study indicates a possible benefit among people at risk for fall accidents, especially for people who are currently experiencing depressive symptoms. Fall accidents prevention should be a daily part for people suffering from depressive symptoms [31]. Positive psychology interventions can contribute to better outcomes in quality of life and physical symptoms [33]. Nevertheless, the related research in this area is still scarce and further research is needed to clarify the functions of such interventions with depressive population.

Our research is conducted based on a large sample size (*N* = 12,527) which can ensure the precision in the odds ratio estimation. However, it should be clarified that individuals whose critical information is missing were not included in this research. Therefore, our study may overestimate the association between depressive symptoms and its subsequent fall accidents. Another major limitation lays in the depressive symptom assessment method. In this research, CES-D score is used to assess depressive symptom. However, CES-D is not the only method for the evaluation of major depressive disorder (MDD) and CES-D may result in underestimation or overestimation for the depressive symptom while other methods are not adopted to assess depressive symptom in CHARLS. As indicated before, nocturia and psychiatric medications used to treat depressive symptoms are associated with higher risk of fall accident; however, psychiatric medications data is not included since only a small number of participants (less than 350) in CHARLS responded this item and CHARLS does not provide nocturia data. This is a major limitation. Besides, our study only focuses on the Chinese population, which may limit its generalizability.

## Conclusions

The results of our research demonstrate that there is a significant association between depressive symptoms and their subsequent fall accidents among Chinese mid-aged and elderly people. The results of this study reveal that elderly people or female people with depressive symptoms have an increased likelihood for fall accidents in their following life. Besides, the results suggest that screening people with depressive symptom may help to prevent fall accidents for the mid-aged and elderly people.

## Abbreviations

ADL: Activities of daily living
AUC: Area under the ROC curve
CES-D: Center for Epidemiologic Studies Depression
CHARLS: China Health and Retirement Longitudinal Study
CI: Confidence interval
GDS: Geriatric depression scale
MDD: Major depressive disorder
OR: Odds ratio
ROC: Receiver operating characteristic curve
SSRI: Selective serotonin reuptake inhibitors
